# Mechanism and Molecular Network of RBM8A-Mediated Regulation of Oxaliplatin Resistance in Hepatocellular Carcinoma

**DOI:** 10.3389/fonc.2020.585452

**Published:** 2021-01-22

**Authors:** Rong Liang, Jinyan Zhang, Zhihui Liu, Ziyu Liu, Qian Li, Xiaoling Luo, Yongqiang Li, Jiazhou Ye, Yan Lin

**Affiliations:** ^1^ Department of Medical Oncology, Guangxi Medical University Cancer Hospital, Nanning, China; ^2^ Department of Experimental Research, Guangxi Medical University Cancer Hospital, Nanning, China; ^3^ Department of Hepatobiliary Surgery, Guangxi Medical University Cancer Hospital, Nanning, China

**Keywords:** RNA-binding motif protein 8A, hepatocellular carcinoma, oxaliplatin, drug resistance, histone deacetylase 9, molecular network

## Abstract

RNA-binding motif protein 8A (RBM8A) is abnormally overexpressed in hepatocellular carcinoma (HCC) and involved in the epithelial-mesenchymal transition (EMT). The EMT plays an important role in the development of drug resistance, suggesting that RBM8A may be involved in the regulation of oxaliplatin (OXA) resistance in HCC. Here we examined the potential involvement of RBM8A and its downstream pathways in OXA resistance using *in vitro* and *in vivo* models. RBM8A overexpression induced the EMT in OXA-resistant HCC cells, altering cell proliferation, apoptosis, migration, and invasion. Moreover, whole-genome microarrays combined with bioinformatics analysis revealed that RBM8A has a wide range of transcriptional regulatory capabilities in OXA-resistant HCC, including the ability to regulate several important tumor-related signaling pathways. In particular, histone deacetylase 9 (HDAC9) emerged as an important mediator of RBM8A activity related to OXA resistance. These data suggest that RBM8A and its related regulatory pathways represent potential markers of OXA resistance and therapeutic targets in HCC.

## Introduction

Hepatocellular carcinoma (HCC) is a highly lethal cancer: it is the fifth most common malignant tumor globally, and its mortality rate ranks third among all cancers (1). The advent of the targeted drug sorafenib opened the door to advanced HCC drug therapies, but first-line therapies are associated with relatively low rates of objective response and progression-free survival (2). Their inefficacy and elevated cost limit their clinical usefulness (3). The complexity of HCC means that it needs to be treated through multiple approaches, including systemic chemotherapy. Oxaliplatin (OXA)-based systemic chemotherapy is a widely used treatment for advanced HCC in Asia, where good efficacy has been achieved (4–6). Nevertheless, chemotherapy resistance has become a tremendous obstacle to the further survival benefit of HCC patients. Identifying the molecules and pathways that give rise to such resistance is critical.

RNA-binding proteins (RBPs) regulate the maturation, translocation, and translation of RNA, making them important in cell development, differentiation, and metabolism (7). We previously showed that the RBP RNA-binding motif protein 8A (RBM8A) is expressed in HCC tumor tissues at higher levels than in normal liver tissues (8, 9). Overexpression of RBM8A is associated with poor overall and progression-free survival in HCC. RBM8A promotes proliferation, migration, and invasion in HCC by activating the epithelial-mesenchymal transition (EMT) (8). Previous studies have shown that EMT is closely related to the promotion of tumor cell metastasis and induction of chemotherapy resistance (10). However, the function of RBM8A in the regulation of chemotherapy resistance remains obscure. More specifically, it has not yet been characterized whether RBM8A is involved in the regulation of OXA resistance *via* initiating EMT in HCC.

The present study explored this hypothesis using a combination of *in vitro* and *in vivo* experiments as well as bioinformatics analyses. Our results identify RBM8A as a potential key factor in OXA resistance in HCC and provide numerous predictions to guide further studies into drug resistance mechanisms.

## Materials and Methods

### Cell Lines and Cell Cultures

Human HCC cell lines (Bel7404, QGY-7703, SMMC-7721, MHCC97L, MHCC97H, HepG2, and SK-HEP-1) and a normal liver cell line (HL7702) were purchased from the Stem Cell Bank of the Chinese Academy of Sciences (Shanghai, China) and were cultured in Dulbecco’s modified Eagle’s medium (DMEM) with 10% fetal bovine serum (FBS; Invitrogen, Carlsbad, CA, USA) in a humidified atmosphere of 5% carbon dioxide at 37°C.

### Establishment of OXA-Resistant HCC Cells

Bel7404 cells were suspended at a density of 1×10^5^ cells/mL, cultured for 24 h, then exposed to an induction dose of OXA (8 μM). After cell growth had stabilized, the drug concentration was increased to 8, 12, 18, 34, 46, 60, 76, 94, 114, and 136 μM. Each dose was maintained for 15 days. Similarly, MHCC97H cells were suspended at a density of 1 × 10^5^ cells/mL, cultured for 24 h, then exposed to an induction dose of OXA (6 μM). After cell growth had stabilized, the drug concentration was increased to 6, 9, 13.5, 20.3, 30.4, 40.5, 55, 70, 86, and 102 μM.

### Establishment of Stable Cell Lines in Which RBM8A Was Overexpressed (OE) or Knocked Down (KD)

Our previous research showed that the short hairpin RNA (shRNA) with the sequence 5’-AGAGCATTCACAAACTGAA-3’ can reduce endogenous levels of RBM8A by more than 80% (8). Using this shRNA, we established two stable KD HCC cell lines, one sensitive to OXA (Bel7404-RBM8A-KD) and one resistant to OXA (Bel7404/OXA-RBM8A-KD). As described in our previous work (8), we obtained two stable OE HCC cell lines, one sensitive to OXA (MHCC97H-RBM8A-OE) and one resistant to OXA (MHCC97H/OXA-RBM8A-OE).

### Total RNA Isolation and Quantitative Real-Time PCR (qRT-PCR)

Total RNA was isolated from parental cell lines (PCLs) and drug resistant (DR)-HCC cells using TRIzol reagent (Invitrogen, USA), then cDNA was reverse-transcribed from 1 mg of total RNA using PrimeScript RT Reagent (TaKaRa, Dalian, China) following the manufacturer’s instructions. Quantitative real-time polymerase chain reaction (qRT-PCR) was performed using SYBR Premix Ex Taq (Takara). PCR primers are described in the **Supplementary Materials and Methods**.

### Protein Extraction and Western Blot Analysis

Western blotting was performed as previously described (8) using antibodies against human RBM8A (catalog no. sc-32312, Santa Cruz Biotechnology, Santa Cruz, CA, USA), human actin (HRP-60008, Proteintech, Rosemont, IL, USA), and rabbit IgG (7074, Cell Signaling Technology, Danvers, MA, USA). Additional reagents are described in the **Supplementary Materials and Methods**.

### Cell Counting Kit-8 Assay

Cell proliferation and half maximal inhibitory concentration (IC_50_) were assessed using the Cell Counting Kit-8 (CCK-8) kit (Dojindo, Japan) according to the manufacturer’s protocol. To measure IC_50_, OXA was added to cultures at concentrations of 40, 80, 320, 640, and 1280 µM, and 48 h later, 10 µL of CCK8 per 100 µL medium was added to the wells. The cells were then incubated at 37°C for another 2 h. Finally, the absorbance was measured at 450 nm using a microplate reader (5082Grodig, Tecan, Austria).

### Flow Cytometry

Cells were collected and stained with an apoptosis detection kit based on phycoerythrin-conjugated annexin V (FXP018-100, 4A Biotech, Beijing, China) according to the manufacturer’s instructions. Apoptosis was analyzed by flow cytometry (FACS Calibur, BD Biosciences, San Jose, CA, USA).

### Wound-Healing Assay, Cell Migration, and Invasion Assays

Detailed methods are described in **Supplementary Materials and Methods**.

### Xenograft Tumorigenesis in Nude Mice

Mouse studies were conducted according to the Guide for the Care and Use of Laboratory Animals and were approved by the Animal Care and Use Committee of the Affiliated Tumor Hospital of Guangxi Medical University, China. BALB/C nude mice (5–6 weeks old, 18–22 g) were randomly divided into two groups of eight mice each. Bel7404/OXA-RBM8A-KD and Bel7404/OXA-NC cells (2 × 10^6^ cells in 100 μL of serum-free DMEM) were injected subcutaneously into nude mice. OXA at 10 mg/kg was injected around the tumor at 1, 2, 4, and 6 weeks after tumor cell injection. The tumor diameter was measured weekly with calipers, and the tumor volume was recorded. After six weeks, the mice were euthanized, and the tumor was removed, weighed, and photographed.

### Immunohistochemical Staining

Hematoxylin & eosin (H&E) staining was performed to assess histopathology of tumors in nude mice, and slides were subsequently stained with a horseradish peroxidase kit (UltraTek, Scytek, Utah, USA) for immunohistochemistry. Immunostaining was performed as described (8) using primary antibodies against RBM8A (same as for western blots), E-cadherin, N-cadherin, Snail, ABCG2, ABCB1, ABCC1, or Ki-67 (9027, Cell Signaling Technology) and reagents from Fuzhou Maixin (Fuzhou, China).

### Whole-Genome Microarrays

Total RNA was isolated from Bel7404/OXA-RBM8A-KD, Bel7404/OXA-RBM8A-NC, MHCC97H/OXA-RBM8A-OE, and MHCC97H/OXA-RBM8A-NC cells using an RNeasy Micro kit (Qiagen, Hilden, Germany) following the manufacturer’s instructions. RNA integrity was assessed using a Bioanalyzer 2100 (Agilent, Santa Clara, CA, USA). Microarray analysis was performed using Affymetrix GeneChip Mouse Genome 430 2.0 Arrays. The arrays were hybridized, washed, and scanned according to the standard Affymetrix protocol. Raw data were normalized using the MAS 5.0 algorithm in GeneSpring 11.0 (Agilent).

### Bioinformatics Analysis

Gene expression was profiled using the limma package in R (11–13). Weighted gene coexpression network analysis (WGCNA) (14) was used to analyze the differential expression profile matrix in cell samples, and gene modules showing coexpression were clustered. Among these module genes, the Clusterprofiler package in R (15) was used to analyze Gene Ontology (GO) functions (p value cutoff = 0.01, q value cutoff = 0.01) and Kyoto Encyclopedia of Genes and Genomes (KEGG) pathways (p value cutoff = 0.05, q value cutoff = 0.2).

Pivot regulators were defined as modulators exerting significant regulation over modules involved in RBM8A-induced resistance. In the pivot analysis, the background set was based on the interaction of transcription factors (TFs) with other proteins in the TRRUST v2 database (16). A network of interactions of long non-coding RNA (lncRNA) and microRNA (miRNA) with protein partners was constructed based on data in the RAID v2.0 database (17). Data on regulation of module genes and pivot TFs by RBM8A were obtained by searching databases with STRING v10.5 (18). The results about pivot regulators and KEGG pathways in the gene module were used to generate a comprehensive map of RBM8A regulation underlying OXA resistance in HCC.

Since qRT-PCR and Western blotting showed histone deacetylase 9 (HDAC9) to be the pivotal TF most closely related to RBM8A-regulated OXA resistance in HCC, the HDAC9-module gene-KEGG signaling pathway was extracted. Finally, a potential mechanism by which the RBM8A-HDAC9 axis regulates drug resistance in HCC was identified.

### Statistical Analyses

Data were analyzed using SPSS 17.0 (IBM, Chicago, IL, USA). All experiments in this study were repeated in triplicate unless otherwise specified. All results were expressed as mean ± standard deviation (SD). Student’s *t* test was used to analyze the statistical significance of differences between groups. Differences associated with *p* < 0.05 were considered significant.

## Results

### Establishment of OXA-Resistant HCC Cell Lines and Analysis of RBM8A Expression

**Figure 1:**
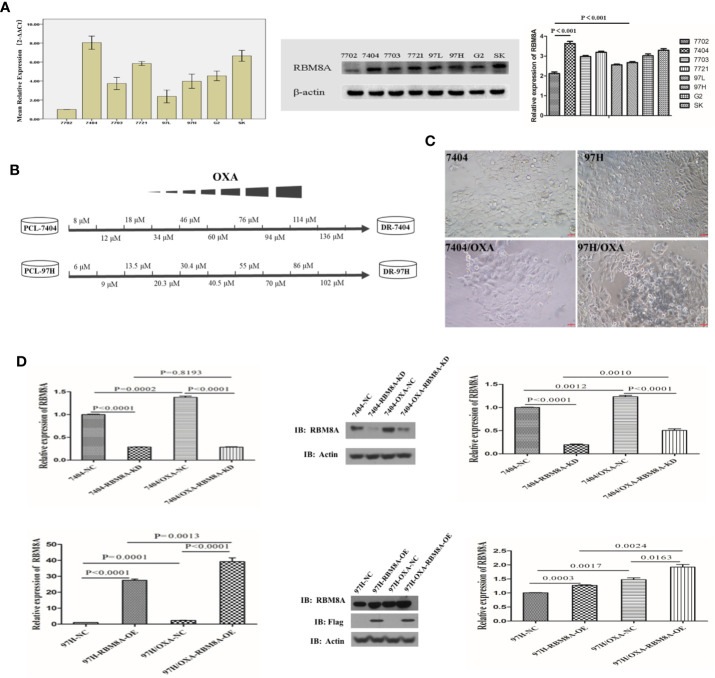
Selection of OXA-resistant hepatocellular carcinoma (HCC) cells and establishment of cell lines in which RBM8A was overexpressed or knocked down. **(A)** Real time (RT)-PCR and western blot analysis of RBM8A expression in HCC cell lines. Western blot results were quantitated. **(B)** Schematic representation of the protocol used to obtain OXA-resistant HCC cells from the parental cell line (PCL). During concentration-elevation and intermittent induction treatment with OXA, each dose was maintained for 15 days. OXA-resistant cell lines were obtained by the end of 6 months. **(C)** Representative phase contrast images of Bel7404 PCLs and drug-resistant cells (DR-HCC cells, *left panels*) or MHCC97H PCLs and DR-HCC cells (*right panels*). Magnification, 20×. Scale bar, 20 μm. **(D)** Knockdown (KD) and overexpression (OE) efficiency of RBM8A in PCLs and DR-HCC cells based on RT-PCR and western blot analysis, compared with the negative control (NC). Western blot data were quantitated (*right panels*). Data were expressed as mean ± SD of three independent experiments, or were representative of three independent observations.

According to the qRT-PCR and Western blotting results, RBM8A showed the lowest expression in the normal human liver cell line HL7702 and was highly expressed in various human HCC cell lines. Among the HCC cell lines, Bel7404 cells showed the highest expression of RBM8A while MHCC97H cells showed the lowest (**Figure 1A**). To discover the potential relationship between RBM8A and oxaliplatin resistance in HCC, we first constructed OXA-resistant HCC cell lines. The schematic representation of the protocol used to obtain OXA-resistant HCC cells from the PCL (**Figure 1B**). The mesenchymal phenotype of DR-HCC cells is shown in **Figure 1C**. Expression of RBM8A was significantly higher in Bel7404/OXA and MHCC97H/OXA cells than in Bel7404 and MHCC97H PCLs, based on qRT-PCR and Western blotting (**Figure 1D**). These results indicates that the expression level of RBM8A may be related to OXA resistance in HCC.

### High RBM8A Expression Promotes Tumor Progression and OXA Resistance of HCC Cells In Vitro

**Figure 2:**
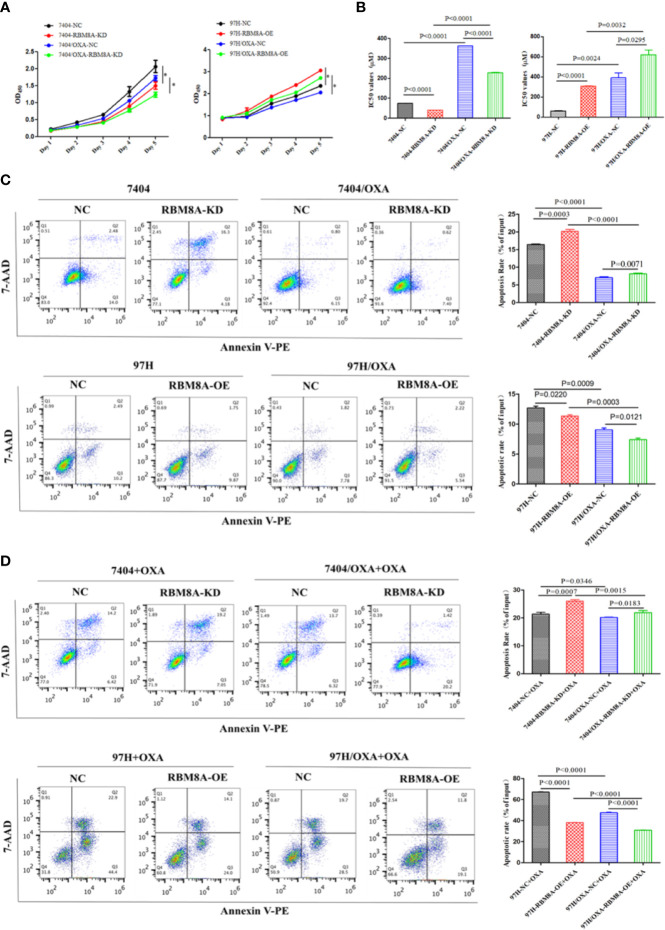
Modulation of RBM8A expression affects proliferation, apoptosis and cell cycle progression in parental cell lines (PCLs) and drug-resistant (DR)-hepatocellular carcinoma (HCC) cells. **(A)** Cell proliferation measured using the Cell Counting Kit-8. *P<0.001. **(B)** Half maximal inhibitory concentration (IC_50_) of oxaliplatin (OXA) when cells were treated for 48 h. **(C)** Apoptosis determined by flow cytometry. Representative quadrant figures were presented on the *left*, and rates of apoptotic PCLs and DR-HCC cells were shown on the *right*. **(D)** Apoptosis in PCLs and DR-HCC cells at 48 h after OXA treatment.

To study the specific role of RBM8A in regulating OXA resistance in HCC cells, we conducted phenotypic studies related to drug resistance. Knocking down RBM8A in Bel7404 cells, which normally express the protein at high levels, significantly reduced proliferation of PCLs and DR-HCC cells. Ectopic expression of RBM8A in MHCC97H cells, which normally express the protein at low levels, significantly enhanced proliferation of PCLs and DR-HCC cells (**Figure 2A**). The IC_50_ of OXA was significantly higher in DR-HCC cell lines than in PCLs. IC_50_ was highest in MHCC97H/OXA-RBM8A-OE cells. Knockdown of RBM8A in Bel7404/OXA cells significantly reduced IC_50_, consistent with the proliferation results (**Figure 2B**). Flow cytometry showed that, regardless of the cell type, the apoptosis level was significantly lower in DR-HCC cell lines than in PCLs (**Figures 2C, D**). In Bel7404 cells with RBM8A knockdown, apoptosis levels were significantly higher in PCLs and DR-HCC cells than in control cells. Conversely, overexpressing RBM8A in MHCC97H cells led to significantly lower apoptosis levels in PCLs and DR-HCC cells than in control cells.

**Figure 3:**
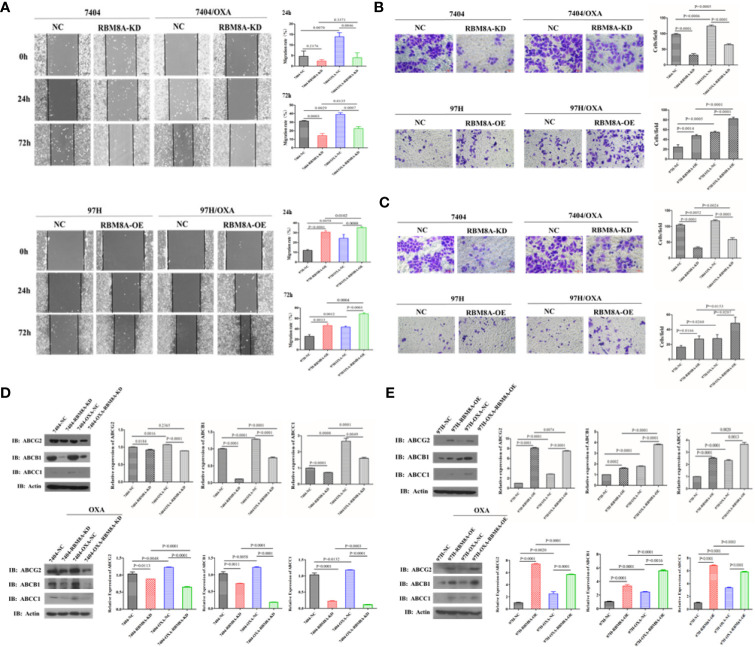
Modulation of RBM8A expression affects the migratory and invasive potential of parental cell lines (PCLs) and drug-resistant (DR)-hepatocellular carcinoma (HCC) cells, as well as the expression of proteins related to drug resistance. **(A)** Wound-healing assay. The scraped areas were photographed at 0, 24, and 72 h after scraping. Migration efficiency was quantitated at 24 and 72 h after scraping (*right panel*). Magnification, 10×. Scale bar, 200 μm. **(B)** Transwell assay. Representative examples of each experimental group are shown. Migration efficiency was quantitated at 24 and 72 h (*right panel*). Magnification, 40×. Scale bar, 50 μm. **(C)** Matrigel-Transwell assay. Representative photographs and quantitation were shown. Data were either representative of three similar observations, or were shown as the mean ± SD of three experiments. Magnification, 40×. Scale bar, 50 μm. **(D)** Western blot analysis of PCL-Bel7404-NC, PCL-Bel7404-RBM8A-KD, DR-Bel7404-NC and DR-Bel7404-RBM8A-KD. Cells were analyzed without OXA treatment (*second row*) or with OXA treatment (*third row*). Data were representative of three similar observations or were shown as the mean ± SD of three experiments. **(E)** Western blot analysis of PCL-MHCC97H-NC, PCL-MHCC97H-RBM8A-OE, DR-MHCC97H-NC and DR-MHCC97H- RBM8A-OE cells as in **(D)**.

In Bel7404 and MHCC97H cells, migration and invasion of the DR-HCC cell lines were significantly greater than those in the corresponding PCLs (**Figures 3A–C**). Drug-resistant Bel7404/OXA-RBM8A-KD cells showed significantly less migration and invasion than drug-resistant Bel7404/OXA-NC cells at 24 and 72 h. Conversely, MHCC97H/OXA-RBM8A-OE cells showed significantly greater migration and invasion than MHCC97H/OXA-NC cells at the same time points.

Overexpression of the ATP-binding cassette (ABC) membrane transport pump is one of the most important contributors to multidrug resistance (19). Thus, we explored the relationship between the expression of RBM8A and that of ABC subfamily G member 2 (ABCG2), ABC subfamily B member 1 (ABCB1) and ABC subfamily C member 1 (ABCC1) in PCLs and DR-HCC cells. Western blotting showed that ABCG2, ABCB1, and ABCC1 levels were significantly higher in Bel7404 and MHCC97H DR-HCC cells than in the corresponding PCLs (**Figures 3D, E**). These three proteins were expressed at significantly lower levels in Bel7404/OXA-RBM8A-KD cells than in Bel7404/OXA-NC cells. Conversely, they were expressed at significantly higher levels in MHCC97H/OXA-RBM8A-OE cells than in MHCC97H/OXA-NC cells. Overall, our data indicate that RBM8A promotes proliferation, migration and invasion of HCC cells, while inhibiting OXA-induced apoptosis.

### High RBM8A Expression Regulates OXA-Resistance *via* EMT in HCC In Vitro

**Figure 4:**
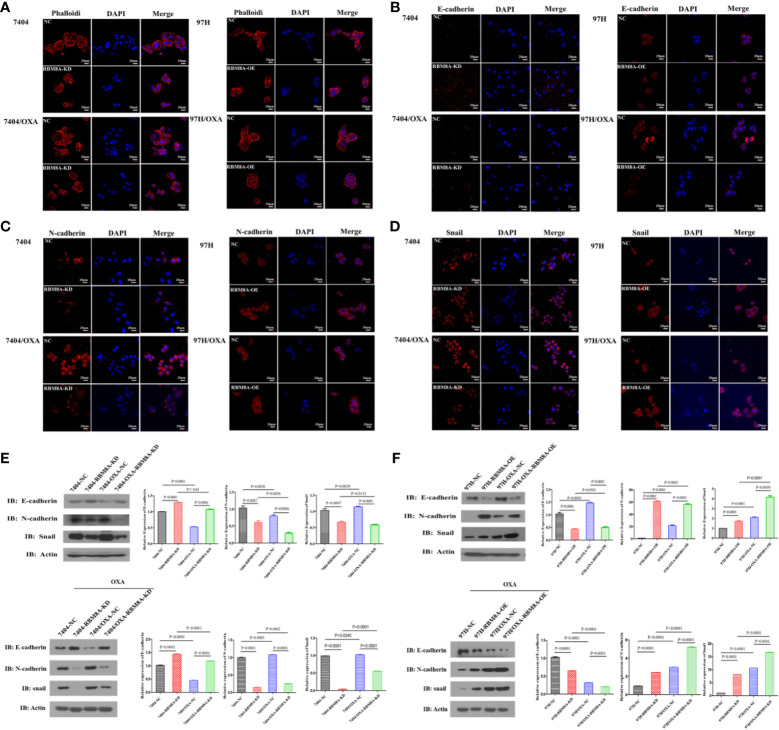
Modulation of RBM8A expression affects the epithelial–mesenchymal transition (EMT) in parental cell lines (PCL) and drug-resistant (DR)-hepatocellular carcinoma (HCC) cells. **(A–D)** Immunofluorescence staining of the **(A)** cytoskeleton, **(B)** E-cadherin, **(C)** N-cadherin, and **(D)** Snail (all red). All confocal microscopy images show the merging with DAPI (blue) in PCLs and DR-HCC cells upon RBM8A knockdown or overexpression. Scale bar, 20 μm. **(E)** Western blot analysis of E-cadherin, N-cadherin, and Snail in PCL-Bel7404 and DR-Bel7404 cells with or without RBM8A knockdown. Cells were analyzed without OXA treatment (*second row*) or with OXA treatment (*third row*). Data were expressed as the mean ± SD of three independent experiments or were representative of three independent observations. **(F)** Western blot analysis of E-cadherin, N-cadherin, and Snail protein expression in PCL-MHCC97H and DR-MHCC97H cells with or without RBM8A overexpression as in **(E)**.

**Figure 5:**
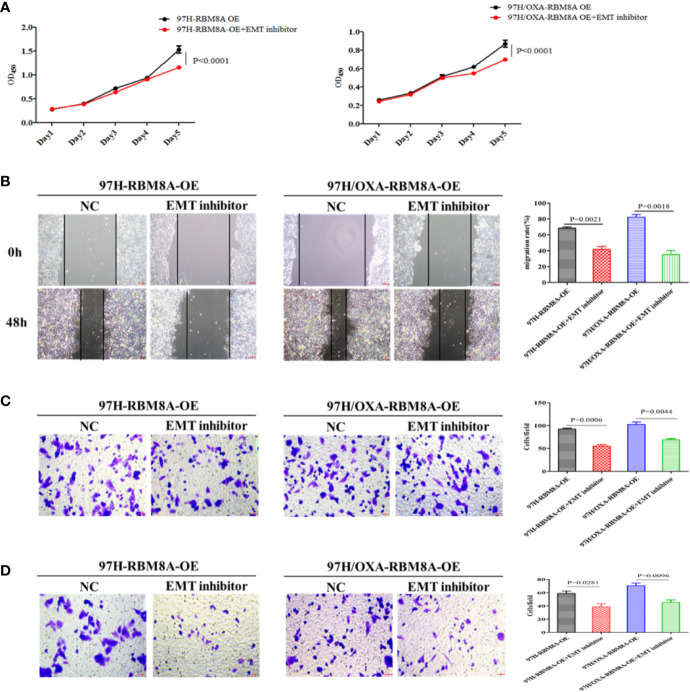
Involvement of the epithelial–mesenchymal transition (EMT) in RBM8A-mediated proliferation, invasion and drug resistance of hepatocellular carcinoma (HCC) cells. **(A)** Cell proliferation was analyzed in PCL-MHCC97H-NC, PCL-MHCC97H-RBM8A-OE, DR-MHCC97H-NC and DR-MHCC97H- RBM8A-OE cells in the presence or absence of the EMT inhibitor C19 using the CCK8 assay. **(B)** Wound-healing assay with or without EMT inhibitor C19. The scraped areas were photographed at 0 and 48 h after scraping. Migration efficiency was quantitated at 48 h after scraping (right). **(C)** Transwell analysis with or without EMT inhibitor C19. **(D)** Matrigel-Transwell analysis with or without EMT inhibitor C19. Magnification, 40×. Scale bar, 50 μm.

Previous reports demonstrate that EMT processes contribute to tumor progression, cancer cell invasion, and therapy resistance (20). Using rhodamine-labeled fluoropeptide to track changes in the cytoskeleton, we found that OXA-resistant Bel7404 and MHCC97H cells were spindle-shaped and exhibited less cell-cell contact than the corresponding PCLs (**Figure 4A**). The ectopic expression of RBM8A in MHCC97H/OXA HCC cells induced loose cell contact and spindle-shaped morphology reminiscent of EMT, whereas RBM8A knockdown in Bel7404/OXA cells resulted in a dramatic shift in the cell morphology from loose cell growth to a tighter cell-cell adherence characteristic of epithelial cells. Furthermore, we sought to determine whether RBM8A levels were associated with epithelial and mesenchymal markers. OXA-resistant Bel7404 or MHCC97H cells showed lower expression of the epithelial protein E-cadherin than the corresponding PCLs, but higher expression of the mesenchymal proteins N-cadherin and Snail (**Figures 4B–D**). Western blotting indicated that MHCC97H/OXA-RBM8A-OE cells, regardless of whether they had been treated with OXA, showed significantly lower levels of epithelial protein E-cadherin but higher levels of mesenchymal proteins N-cadherin and Snail than MHCC97H/OXA-NC cells. Conversely, Bel7404/OXA-RBM8A-KD showed significantly higher levels of E-cadherin and lower levels of N-cadherin and Snail than Bel7404/OXA-NC cells (**Figures 4E, F**). Further suppression of the EMT pathway using the EMT inhibitor C19 significantly reversed the proliferation, invasion and migration of RBM8A-enhanced PCLs and DR-HCC cells (**Figures 5A–D**). Taken together, these results and our previous studies indicate that the EMT pathway is one of the important mechanisms by which RBM8A regulates the malignant phenotype and OXA resistance of HCC.

### RBM8A Regulates OXA Resistance in HCC Xenograft Models *via* the EMT

**Figure 6:**
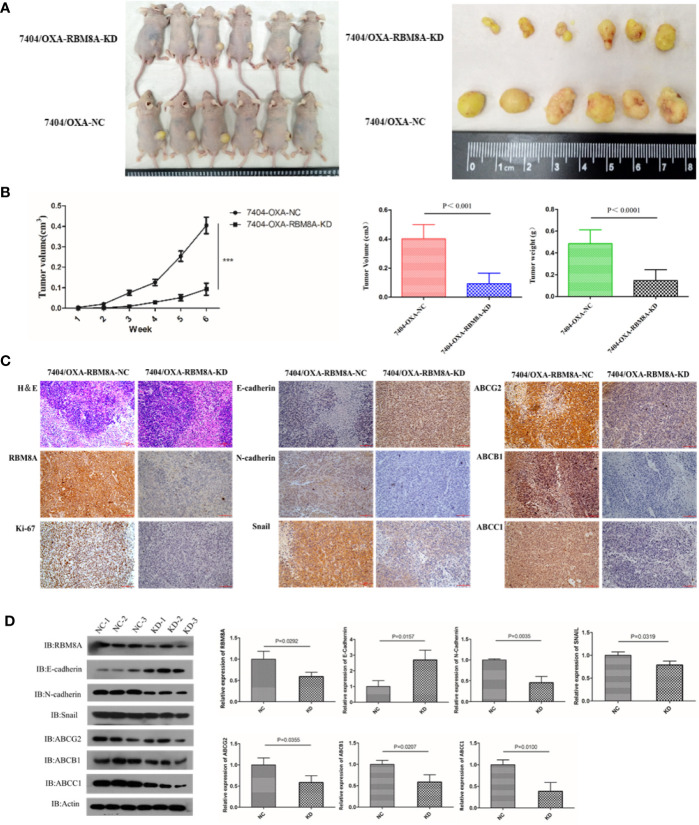
Effects of RBM8A on drug-resistant (DR)-hepatocellular carcinoma (HCC) tumorigenesis *in vivo*. **(A)** Bel7404/OXA-RBM8A-KD and control cells were injected orthotopically into mammary fat pads of nude mice, which were then injected with OXA at 10 mg/kg around the tumor at 1, 2, 4, and 6 weeks. The growth of tumors was followed during a six-week period. Photographs of primary tumors are shown on the *right*. **(B)** Comparison of tumor volume in Bel7404/OXA-RBM8A-KD and Bel7404/OXA-RBM8A-NC animals. Mice injected with Bel7404/OXA-RBM8A-KD cells formed smaller (p < 0.0001) and lighter (p = 0.0004) tumors than mice injected with control cells (NC). ***<0.001 **(C, D)** Immunohistochemical staining and western blotting of Bel7404/OXA-RBM8A-KD and Bel7404/OXA-RBM8A-NC tumors. Data were expressed as the mean ± SD of three independent experiments or were representative of three independent observations. Magnification, 20×. Scale bar, 100 μm.

To evaluate *in vivo* the ability of RBM8A to promote OXA resistance in HCC through the EMT, nude mouse xenograft models were established using Bel7404/OXA-NC and Bel7404/OXA-RBM8A-KD cells. Tumor size, tumor formation rate, and body weight were lower in Bel7404/OXA-RBM8A-KD animals than in control mice (**Figures 6A, B**). Compared to Bel7404/OXA-RBM8A-NC tumors, Bel7404/OXA-RBM8A-KD tumors expressed lower levels of Ki-67, ABCG2, ABCB1, ABCC1, and the mesenchymal proteins N-cadherin and Snail, but higher levels of the epithelial protein E-cadherin (**Figures 6C, D**). These data suggest that the reduction of RBM8A expression inhibits HCC growth and EMT processes, sensitizing HCC to OXA *in vitro*.

### RBM8A Regulates the Transcription of Genes in OXA Resistance in HCC *via* a Network Involving Tumor-Associated TFs, ncRNAs, and Signaling Pathways

#### Expression of Dysregulated Molecules Associated With RBM8A in OXA-Resistant HCC

The flow chart of the bioinformatics analysis is shown in **Supplementary Figure 1A**. Wayne mapping identified 8365 genes differentially expressed between Bel7404/OXA-NC and Bel7404/OXA-RBM8A-KD cells, as well as between MHCC97H/OXA-NC vs. MHCC97H/OXA-RBM8A-OE cells (**Supplementary Figure 1B**). These genes may be associated with RBM8A-mediated OXA resistance in HCC (**Supplementary Table S1**). WGCNA of these differentially expressed genes revealed patterns of coexpression that we were able to organize into five modules of OXA resistance-related genes in HCC (**Supplementary Figures 1C–E**). Based on the association between gene modules and cells, we found that the fourth module positively correlated the most strongly with the Bel7404/OXA-RBM8A-KD phenotype, while the third module positively correlated strongly with the MHCC97H/OXA-RBM8A-OE phenotype (**Supplementary Figures 1F, G**).

#### Identification of the Biological Molecular Network of RBM8A in OXA-Resistant HCC

Exploring the functions and pathways involved in the relevant modules helps to establish molecular bridges between gene modules and disease pathology and pharmacology, potentially deepening understanding of the molecular mechanism. Therefore, we analyzed the enrichment of GO biological processes and KEGG pathways in the five modules. From these results, we found that the potential functions of genes in the five modules were mainly related to mRNA splicing, ribonucleoprotein complex biogenesis, and ncRNA processing (**Supplementary Table S2**). RBM8A-related genes were involved mainly in the following KEGG pathways: PI3K-Akt signaling, MAPK signaling, viral carcinogenesis, mRNA surveillance, and cell cycle (**Supplementary Table S3**).

**Figure 7:**
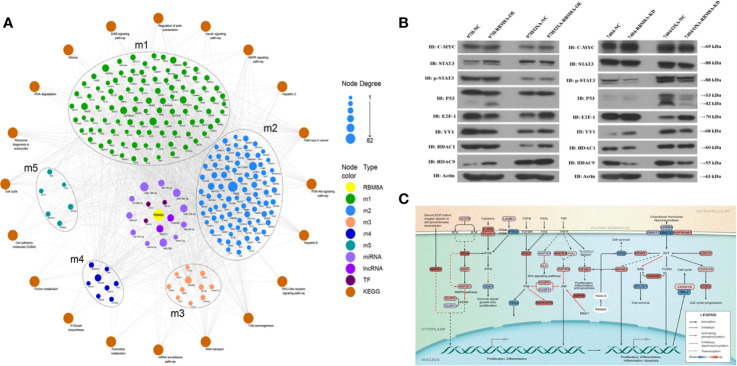
Molecular network showing how RBM8A may regulate oxaliplatin (OXA) resistance in hepatocellular carcinoma (HCC). **(A)** Bioinformatics analysis integrating the regulatory information of RBM8A on module genes and pivot factors to construct a comprehensive overview of RBM8A-mediated OXA resistance in HCC. In this landscape, long non-coding RNAs (lncRNAs), microRNAs (miRNAs), and transcription factors (TFs) mediate the ability of RBM8A-regulated module genes and their downstream signaling pathways to confer drug resistance on HCC cells. **(B)** Western blot analysis of the expression of transcription factors MYC, STAT3, P53, E2F1, YY1, HDAC1, and HDAC9 in HCC cell lines. Western blotting revealed that, after overexpression or knockdown of RBM8A in parental cell lines (PCLs) and drug-resistant (DR)-HCC cells, HDAC9 expression regulated by RBM8A was associated with OXA resistance in HCC cells. **(C)** Bioinformatics analysis combined with quantitative real time PCR (qRT-PCR) and western blotting revealed that HDAC9 is the pivotal transcription factor most closely related to the RBM8A-mediated regulation of OXA resistance in HCC. The HDAC9-module gene-KEGG signaling pathway was extracted, and the potential mechanism by which the RBM8A-HDAC9 axis regulates drug resistance in HCC was identified.

We used TF- and ncRNA-targeting regulatory genes as a background set for hypergeometric prediction analysis. The results identified 1663 ncRNAs and 38 TFs with regulatory influence over module genes, which we considered candidate pivotal regulators (**Supplementary Tables S4** and **S5**). Among them, MALAT1, MYCN, HDAC9, FENDRR, and other key regulatory nodes showed significant regulatory influence over more than one module and thus were identified as core pivot regulators. These core pivot regulators may be driven by RBM8A and may regulate genes and pathways related to OXA resistance in HCC. Based on the genes within the modules and the KEGG signaling pathways, we obtained a comprehensive map of RBM8A regulation of OXA resistance in HCC (**Figure 7A**).

Combining the WGCNA and hypergeometric predictions, we selected the following pivotal regulators with significant effects on the module genes: the lncRNAs MALAT1 and FENDRR, and the TFs MYC, STAT3, P53, E2F1, YY1, HDAC1, and HDAC9. qRT-PCR and Western blotting were used to verify the correlation between RBM8A and core pivotal regulator expression in HCC cell lines *in vitro* (**Figure 7B** and **Supplementary Figure 2**). HDAC9 expression was significantly higher in DR-HCC cells than in PCLs, and in both cell types, it was up- or down-regulated after RBM8A was overexpressed or knocked down, respectively. Thus, HDAC9 is closely related to RBM8A-regulated OXA resistance in HCC cells.

Based on the proposed downstream signaling network involving RBM8A and HDAC9 (**Figure 7C**), NFKB1 and TP53 are predicted to be direct target genes of HDAC9. In addition to the NRAS oncogene, several cyclin-dependent kinase and MAPK family genes may also be involved. Enrichment analysis suggests that the module genes regulated by the RBM8A-HDAC9 axis participate mainly in the PI3K-Akt and MAPK pathways, which help control cell proliferation, inflammation, apoptosis, and the cell cycle.

## Discussion

RBM8A (also known as Y14) was identified only within the last decade and has since been shown to play roles in the formation, degradation, translation, and quality control of mRNA as a core component in the exon junction complex (21, 22). Abnormal expression of RBM8A may play an important role in activating signal transduction pathways that drive oncogenesis (9, 23). We found that RBM8A overexpression promoted proliferation, reduced apoptosis and increased the chemotherapeutic resistance of HCC cells to OXA, while RBM8A knockdown reversed these effects, consistent with reports that the deletion of the RBM8A gene down-regulates Bcl-Xs, Bim, and Mcl-1, as well as several proapoptotic genes, including members of the Bcl-2 family, thereby inducing apoptosis (24).

Malignant tumors are often resistant to antitumor drugs, they show unlimited proliferative ability, and they eventually progress to local infiltration and distant metastasis (25). In our study, RBM8A overexpression further increased the migration and invasion of HCC cells, and this involved the promotion of the EMT, which is the first step in invasion and metastasis (20, 26). Consistently with our work, a previous study (27) reported that OXA-resistant HCC cell lines showed higher incidence of a mesenchymal phenotype.

How HCC cells become resistant to OXA is complex. Several mechanisms have been proposed, including apoptosis escape, autophagy activation, drug excretion, and enhanced epigenetic transformation (28–32). Inactivation of multiple signaling pathways is thought to alter expression of genes involved in apoptosis and proliferation to confer resistance, and several cytokines also control one another through regulatory networks. The EMT process is also central to most models of drug resistance (25, 33–35). In order to take into account these multi-dimensional interactions, a comprehensive analysis combining experimental and bioinformatics approaches is needed. Using such an approach, we identified several TFs and ncRNAs as well as their corresponding metabolic pathways that may help RBM8A regulate OXA resistance in HCC (**Figure 6** and **Supplementary Tables S4** and **S5**).

Several of these TFs and ncRNAs have already been implicated in HCC growth and drug resistance, validating our approach. In MHCC97H/OXA cells, expression of most genes involved in cell death or apoptosis (including Ras, MAPK, and p53 pathway genes) is altered relative to OXA-sensitive cells (36), and genes encoding TFs and kinases are the most up-regulated. The ncRNAs miR-125 (35), miR-31 (37), H19 (38), and NR2F1 (39) have been linked to the development and progression of HCC and drug resistance. NF-κB, PI3K/Akt, GSK3β/β-catenin, and HIF-1α signaling pathways have also been implicated in HCC chemoresistance (40–43).

We identified and validated HDAC9 as a key TF that likely helps RBM8A regulate OXA resistance in HCC. Abnormally high HDAC9 expression is closely related to proliferation, invasion, and metastasis of various tumor types (44–48), and it may up-regulate genes that participate in the oncogenic Ras, VEGF, MAPK, and EGFR signaling pathways (49). HDAC9 is known to regulate the transcription of tumor suppressor gene p53 (47), deacetylated FoxO1 (50), SOX9 (51), and transcriptional coactivator with PDZ-binding motif (TAZ) (52). Changes in HDAC inhibitors show promise as anticancer treatments (53, 54). Our study is one of the few to analyze HDAC9 in the context of HCC. Given that previous work has shown that HDAC9 can down-regulate miR-376a and thereby promote cancer (55), future studies should identify genes, miRNAs and ncRNAs targeted by HDAC9 in drug-resistant HCC.

## Conclusions

Our study shows that RBM8A can induce EMT in HCC cells, thereby affecting proliferation, apoptosis, migration, and invasion, as well as promoting OXA resistance. Gene array combined with bioinformatics analysis revealed that RBM8A has a wide range of transcriptional regulatory capabilities in drug-resistant HCC, including the ability to regulate several important tumor-related signaling pathways. In particular, HDAC9 was identified as an important mediator of RBM8A-induced OXA resistance. These data suggest that RBM8A and its related regulatory pathways represent potential markers of OXA resistance and potential therapeutic targets in HCC.

## Data Availability Statement

The raw data supporting the conclusions of this article will be made available by the authors, without undue reservation.

## Ethics Statement

The animal study was reviewed and approved by Animal Care and Use Committee of the Affiliated Tumor Hospital of Guangxi Medical University.

## Author Contributions

RL, JY, and YL came up with the design and conception. The data analysis and visualization were conducted by JZ, ZHL, ZL, QL, XL, and YL. The original writing of the draft and its editing were by JY and YL. All authors contributed to the article and approved the submitted version.

## Funding

This research was supported by the National Natural Science Foundation of China (NO. 81660498, 82060427 and 81803007), Guangxi Key Research and Development Plan (NO. GUIKEAB19245002), Guangxi Scholarship Fund of Guangxi Education Department, General Program of Guangxi Natural Science Foundation (NO. 2020GXNSFAA259080), Youth Talent Fund Project of Guangxi Natural Science Foundation (NO. 2018GXNSFBA281030, 2018GXNSFBA281091), Guangxi Medical and Health Appropriate Technology Development and Application Project (No. S2017101, S2018062), Guangxi Medical University Training Program for Distinguished Young Scholars, Science and Technology Plan Project of Qingxiu District, Nanning (NO. 2020037, 2020038).

## Conflict of Interest

The authors declare that the research was conducted in the absence of any commercial or financial relationships that could be construed as a potential conflict of interest.
