# Supplementary material for: Mechanism and Molecular Network of RBM8A-Mediated Regulation of Oxaliplatin Resistance in Hepatocellular Carcinoma
Source: Front Oncol. 2021 Jan 22;10:585452. doi: 10.3389/fonc.2020.585452 (PMC7862710; doi:10.3389/fonc.2020.585452)
Supplement: Supplementary file 1 [file DataSheet_1.zip › ▓╣│Σ▒φ/The supplementary material for Materials and methods7.5.docx]

**Supplementary Materials and Methods**

***Quantitative real-time PCR (qRT-PCR) analysis***

The following PCR primers were used:

RBM8A-F, GCGTGAGGATTATGACAGCGTG

RBM8A-R, TTCGGTGGCTTCCTCATGGACT

human MALAT1-F, TTGTAGACTGGAGAAGATAGG

human MALAT1-R, ACTGAAGAGCATTGGAGAT

human FENDRR-F, CCTTGCCACCTCAGCGCT

human FENDRR-R, AGGCCATAGGTTGTTCAGGGTC

human MYC-F, CCTGGTGCTCCATGAGGAGAC

human MYC-R, CAGACTCTGACCTTTTGCCAGG

human HDAC1-F, ATTTGCTGCTCAACTATGGTCTCTA

human HDAC1-R, TGCCACAGAACCACCAGTAGA

human HDAC9-F, TCTCGTCTCCAGGACTCACTCT

human HDAC9-R, GCACTGGTGTTTCAGCATCAAGG

human STAT3-F, CTTTGAGACCGAGGTGTATCACC

human STAT3-R, GGTCAGCATGTTGTACCACAGG.

human P53-F, CCTCAGCATCTTATCCGAGTGG

human P53-R, TGGATGGTGGTACAGTCAGAGC

human E2F1-F, ACGCTATGAGACCTCACTGAA

human E2F1-R, TCCTGGGTCAACCCCTCAAG

human YY1-F, ACGGCTTCGAGGATCAGATTC

human YY1-R , TGACCAGCGTTTGTTCAATGT

Quantification was normalized to β-actin, which served as the internal control. The relative mRNA expression was calculated using the 2-ΔΔCt method.

***Antibodies for western blot detection:***

anti-human ABCG2 (Cell Signaling Technology, 42078), anti-human ABCB1 (Cell Signaling Technology, 13978S), anti-human ABCC1 (Cell Signaling Technology, 72202), anti-human E-cadherin (Cell Signaling Technology, 14472), anti-human N-cadherin (Cell Signaling Technology, 13116), anti-human Snail (Cell Signaling Technology, 3879), anti-human C-MYC (Sigma, C3956), anti-human HDAC1 (Abways, CY5890), anti-human HDAC9 (Abways, CY5154), anti-human STAT3 (Abways, CY5292), anti-human p-STAT3 (CST, 9145S), anti-human P53 (Santa, Sc-126), anti-human E2F1 (CST, 3742S), anti-human YY1 (Abways, CY6572), EMT inhibitor 1 (MCE, HY-101275).

***Wound-healing assay***

Parental cell lines (PCLs) and drug-resistant hepatocellular carcinoma (DR-HCC) cells were seeded in a 24-well plate and cultured in an incubator until 100% confluence. A pipette tip was scraped from the center of the well to the lower end of the well plate to form a scratch. After the scraped cells were washed away, the adherent cells were incubated in serum-free medium. An image was taken at 0 h as a control, and then the plate was placed in an incubator with a 5% CO_2_ atmosphere. The plates were removed at 24, 48, and 72 h after scratching to obtain photographs under a fluorescence microscope (200×).

***Cell*** ***migration and invasion assays***

Proteins related to the cytoskeleton and epithelial–mesenchymal transition (EMT) in PCLs and DR-HCC cells were stained by immunofluorescence. PCLs and DR-HCC cells were suspended on a sterilized coverslip, and were removed after 24 h of incubation in the incubator. The cells were fixed with 4% paraformaldehyde at room temperature, and 0.5% Triton X-100 (Shanghai Shenggong Biological, China) was added to permeabilize the cells. Next, 200 μL of TRITC-labeled phalloidin (Sigma), 200 μL of 100 nM DAPI (Gibco, USA) (for counterstaining nuclei), and fluorescence mounting medium (Gibco, USA) were added to the slides. Laser confocal microscopy (Olympus) was performed with a TRITC excitation/emission filter (Ex/Em = 545/570 nm) and a DAPI excitation/emission filter (Ex/Em = 364/454 nm). Slides were treated with the same primary antibodies as those used in the western blot experiments, and Alexa Fluor® 488-conjugated donkey anti-rabbit IgG (H+L) secondary antibody (1:1000) was purchased from Invitrogen (USA) and incubated with the slides at 37 °C for 1 h before DAPI counterstaining.
