# Supplementary material for: Mechanism and Molecular Network of RBM8A-Mediated Regulation of Oxaliplatin Resistance in Hepatocellular Carcinoma
Source: Front Oncol. 2021 Jan 22;10:585452. doi: 10.3389/fonc.2020.585452 (PMC7862710; doi:10.3389/fonc.2020.585452)
Supplement: Supplementary file 2 [file DataSheet_2.zip › ▓╣│Σ═╝/Supplementary figure legends 7.5.docx]

**Supplementary figures legends**

**Figure S1. Organization of differentially expressed genes into modules based on coexpression.**

(A) Schematic representation of the biological information analysis used in this study. WGCNA, weighted gene coexpression network analysis; GO, Gene Ontology; KEGG, Kyoto Encyclopedia of Genes and Genomes; TF, Transcription factor; ncRNA, noncoding RNA.

(B) Venn diagram showing the intersection of differentially expressed genes between Bel7404/OXA-NC vs. Bel7404/OXA-RBM8A-KD cells (blue circle) and MHCC97H/OXA-NC vs. MHCC97H/OXA-RBM8A-OE cells (yellow circle).

(C) Analysis of intersecting genes identified five modules based on their coexpression. Each color of the X-axis represents a module, and the Y-axis represents the weight of the corresponding module.

(D) Heat map of the coexpressed gene modules. Expression of genes within the same module show similar expression, while genes in different modules show different expression.

(E) Cluster tree diagram of the gene modules. Among them, the circle color corresponds to the module color, while the adjacent circle represents the tree-like neighbor relationship between the two modules.

(F) Correlation heat map between gene modules. The XY axes represent the five different modules, and the color along the XY axes corresponds to the correlation between the modules from 1 (strong, red) to 0 (weak, blue).

(G) Relationship heat map of module phenotypic characteristics. The X-axis represents different HCC cell lines, while the Y-axis represents the five coexpression modules. The box color along the XY axes corresponds to intermodule correlation from 1 (positive, red) to -1 (negative, blue). The numbers in the box represent the relevance score.

**Figure S2.** **qRT-PCR analysis of the long non-coding RNAs (lncRNAs) MALAT1 and FENDRR and the transcription factors MYC, STAT3, P53, E2F1, YY1, HDAC1, and HDAC9 in hepatocellular carcinoma (HCC) cell lines.**

(A) After RBM8A was overexpressed in MHCC97H parental cell lines and drug-resistant cell lines, qRT-PCR analysis of the lnc MALAT1 and MYC, STAT3, P53, E2F1, YY1, HDAC1, HDAC9 expression.

(B) After RBM8A was knocked down in Bel7404 parental cell lines and drug-resistant cell cells, qRT-PCR analysis of the lnc MALAT1 and FENDRR and MYC, STAT3, P53, E2F1, YY1, HDAC1, HDAC9 expression.

**Supplementary tables legends**

**Table S1. Intersection of differentially expressed genes associated with RBM8A in OXA-resistant HCC.**

**Table S2. Biological processes involving modular genes based on Gene Ontology analysis.**

**Table S3. Signaling pathways involving modular genes.**

**Table S4. Non-coding RNAs (ncRNAs) regulating modular genes.**

**Table S5. Transcription factors (TFs) regulating modular genes.**
