# Supplementary figures and images for: Mechanism and Molecular Network of RBM8A-Mediated Regulation of Oxaliplatin Resistance in Hepatocellular Carcinoma
Source: Front Oncol. 2021 Jan 22;10:585452. doi: 10.3389/fonc.2020.585452 (PMC7862710; doi:10.3389/fonc.2020.585452)

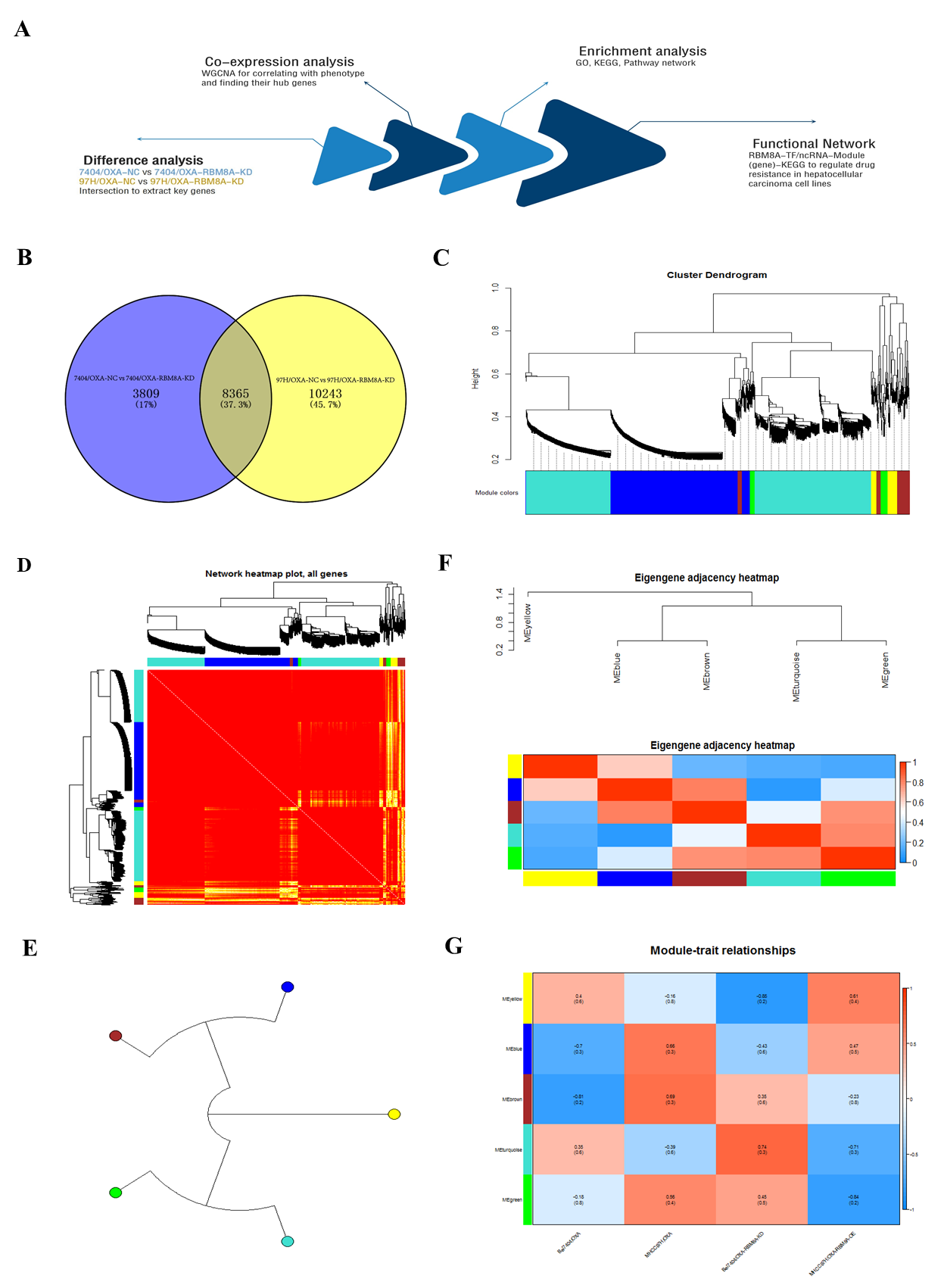

Supplement: Supplementary file 2 [file DataSheet_2.zip › ▓╣│Σ═╝/Fig S1.tif]

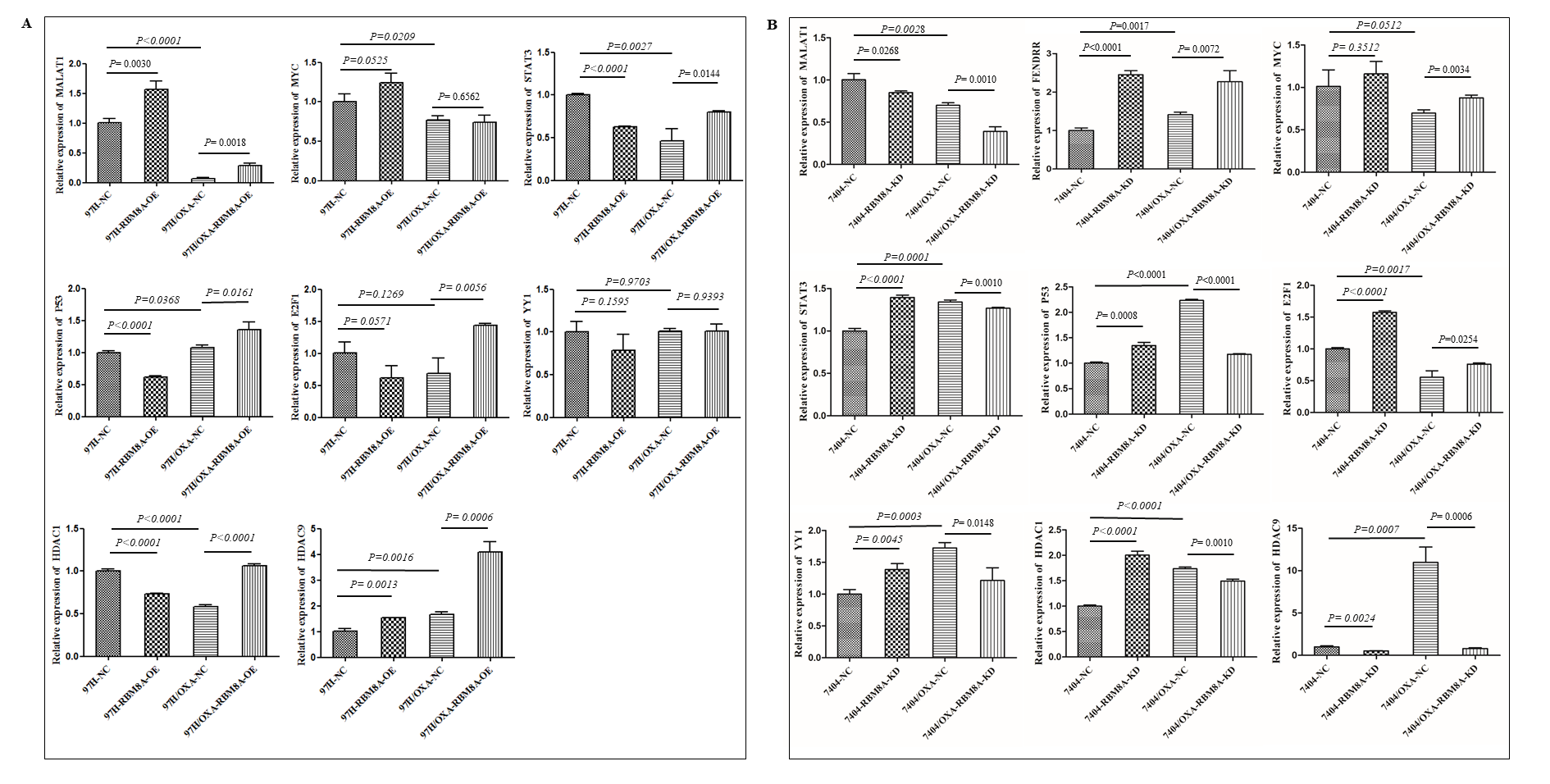

Supplement: Supplementary file 2 [file DataSheet_2.zip › ▓╣│Σ═╝/Fig S2.tif]
